# Factors that influence inter-organisational integration: a qualitative exploration of service providers’ perspectives from an integrated care initiative

**DOI:** 10.1186/s12913-025-13051-7

**Published:** 2025-07-10

**Authors:** Fani Liapi, Angel Marie Chater, Gurch Randhawa, Amy Stephenson, Yannis Pappas

**Affiliations:** 1 Institute for Health Research, University of Bedfordshire, Luton, LU2 8LE UK; 2 Institute for Sport and Physical Activity Research, University of Bedfordshire, Bedford, MK41 9EA UK; 3 University College London, Centre for Behaviour Change, London, WC1E 7HB UK; 4 Turning Point, London, E1 8AA UK

**Keywords:** Integrated care, Inter-organisational, Barriers, Facilitators, Implementation

## Abstract

**Background:**

Integrated care uses inter-professional and inter-organisational collaboration to ensure quality care for those with complex healthcare needs. Whilst inter-organisational collaboration is seen as a facilitator of integration, it has its own complexities and challenges. This study sought to investigate barriers to and facilitators of inter-organisational integration between the partnered organisations of an integrated care initiative in Luton, UK, as perceived by healthcare professionals.

**Methods:**

Face to face semi – structured interviews were conducted between November 2019 and March 2020 with twenty service providers of an integrated service for physical and mental health. Thematic analysis was used to explore the experiences and perceptions of service providers on the integration of healthy lifestyle and mental health services of “Total Wellbeing Luton”.

**Results:**

Five primary themes were identified: (1) Culture, (2) Communication Structures, (3) Strategic alignment, (4) Workforce dynamics, and (5) Expectation and reality of the integration. The majority of the identified themes reflect factors that facilitate or impede the integration between the participating organisations. Excellent relationships between healthcare professionals and staff’s motivation have been identified as facilitators to inter-organisational integration, while high staff turnover, lack of shared IT system and absence of co-location have been identified as barriers. Also, a theme reveals that often there was a discrepancy between the expected and the actual implementation and outcomes of integration.

**Conclusions:**

As a part of a larger evaluation programme, this study attempts a comprehensive understanding of the inter-organisational integration in the specific context of Total Wellbeing Luton. The findings and recommendations can support the inter-organisational integration of current and future integrated care initiatives in the UK and elsewhere.

**Supplementary Information:**

The online version contains supplementary material available at 10.1186/s12913-025-13051-7.

## Background

Obesity is a global public health concern with significant economic implications, with 63.8% of adults in England being either obese or overweight, as reported in the 2021 Health Survey [1, 2]. Obesity is linked to several mental health conditions, like depression and anxiety disorders [3–8], making mental ill health the single largest cause of disability in the UK [9]. Obesity and mental health disorders are recognised as complex public health issues caused by a variety of influences, such as genetics, environment, and psychosocial factors [10–12]. The increased prevalence and complexity of obesity and mental health disorders have reshaped the focus of healthcare systems worldwide; from acute care to developing care models that emphasise continuity of care, integrated services, prevention through patient education, and self-management support [13].

In World Health Organisation’s (WHO) guidelines, integrated care programmes are recommended to ensure quality care for those with complex healthcare needs by coordinating and securing continuity of care [14]. Integrated care models combine clinical, and administrative practices across the health and social care sectors to provide complex, multi-component interventions [15–17]. To ensure continuity and provide comprehensive care, inter-professional and inter-organisational collaboration are key elements of integrated care [18].

There is one definition that is widely used within the literature [19–21] and defines ‘integration’ and ‘integrated care’ as:

*Integration is a coherent set of methods and models on the funding*,* administrative*,* organisational*,* service delivery*,* and clinical levels designed to create connectivity*,* alignment*,* and collaboration within and between the cure and care sectors. The goal of these methods and models is to enhance quality of care and quality of life*,* consumer satisfaction*,* and system efficiency for patients with complex*,* long-term problems cutting across multiple services*,* providers*,* and settings. The result of such multi-pronged efforts to promote integration for the benefit of these special patient groups is called ‘integrated care’.*

There are references for seven main types of integration in the literature: functional, organisational, professional, clinical, service, systemic, and normative [22–27]. In terms of the breadth of integration, these types of integration can occur in ways that have been described as horizontal, when similar organisations at the same stage in the process of service delivery are joined together (e.g. mergers of acute hospitals) or vertical integration, when different organisations at different levels of care (e.g., primary, secondary, and tertiary) are combined under one management umbrella [24–26, 28, 29]. The Rainbow Model of Integrated Care, developed by Valentijn et al., organises these features into six dimensions: service delivery, leadership and governance, workforce, financing, technologies, and medical products, and information and research [17]. This work emphasises the importance of integrated care, suggesting that it should be implemented at various system levels to ensure continuous, coordinated service provision to individuals and populations. To improve the quality of life of people with physical and mental health comorbidities, there is a need for integration across physical and mental health services. In the UK, there is a policy commitment to reshape healthcare services and to develop integrated care models [30, 31]. Integrated care initiatives aim to make services streamlined, encourage different services to work together, and provide continuity of care [32]. Systematic reviews have provided evidence of the improvement of patients’ health outcomes and cost-effectiveness in integrated care [33–37]. This justifies why integrated care has to become an essential part of the health system to respond effectively to those needing treatment [38].

Since 1997, inter-organisational partnerships have been promoted to improve healthcare services and transform them, ensuring seamless care for patients with complex healthcare needs who receive a mix of services provided from different organisations [21, 39–42]. Inter-organisational collaboration arrangements are a key focus area of healthcare planning and policy worldwide [43–45]. For over three decades, in the UK, the NHS has trialled the integration of health and social care partners, such as the Vanguard initiatives [41]. New Care Model Vanguards was launched in 2015, intending to support the vision for the future development of the NHS which could be focused upon new ways of working to improve care delivery [46]. The ‘Vanguards’ sites operated at place level as they served populations between 250-500k [47] which could subsequently be replicated across England [48]. In 2018, the Vanguard programme ended, and the oversight of new care models was assigned to NHS England’s new System Transformation Group (STG), focusing on developing integrated care systems (ICSs), as a more advanced form of sustainability and transformation partnerships (STPs) [49].

The existing body of research on the impact and effectiveness of integrated care suggests that improved quality of care, increased patient satisfaction, and improved access to care are some of the positive outcomes of integrated care [42, 50–52]. Despite these positive outcomes, integration provides opportunities for multi-level partnerships within and across organisations. The process of inter-organisational collaboration has its own complexities and challenges, such as working around agendas and conflicts, building trust, and navigating communication pathways [53]. Therefore, as a consequence of implementing inter-organisational partnerships, workforce practices of healthcare professionals, become significantly more complex, and substantial effort, investment of time, and financial resources are required [53, 54].

Previous research has found that a high level of collaboration between the care partners is essential to achieve improved quality of service delivery and patients’ health outcomes [52, 55]. Attention has been paid to the healthcare staff’s perceptions about the delivery of integrated care [56, 57]. Existing research has examined the experiences and perceptions of healthcare professionals of integrated care initiatives and inter-organisational integration [58, 59]. Integrated care is an approach that aims to improve the coordination and delivery of healthcare services across different organizations and settings, to provide more patient-centered and efficient care.

Healthcare professionals’ experiences with integrated care initiatives and inter-organisational integration vary significantly. Healthcare professionals recognise significant utility in these models due to enhanced communication, ease of implementation, and improved patient-centered care outcomes [60, 61]. Facilitating factors such as integrated leadership, multi-way communication, and the ability to contribute to multiple working groups enhance professional acceptance and participation [60]. Additionally, mutual trust, clear working routines, and improved linkages between organizations are seen as empowering features [61]. Achieving successful integration of care necessitates a clear understanding and collaboration with community organizations and local healthcare practitioners [62]. Despite these advantages, substantial barriers remain such as diverse IT structures, resistance to change, and lack of time to introduce changes [60]. Furthermore, organizational culture and traditional care models within hospitals further impede integration efforts [61].

The present study was commissioned to evaluate the “Total Wellbeing Luton” a new integrated care service in Luton, UK after its first year of delivery. Two organisations, Turning Point and Active Luton partner to provide integrated psychological therapies and healthy lifestyle services, aiming to create a proactive continuum of care for Luton residents. The study is grounded in the Complex Adaptive System (CAS) theory, which offers a framework for understanding the complexities of integrated healthcare services and identifying the requirements for achieving high levels of integration. By rejecting formal hierarchies and emphasizing holistic thinking, CAS theory helps to identify potential challenges and solutions within the Luton integrated care initiative. This study focuses on exploring the experiences and perceptions of professionals involved in the implementation and delivery of the integrated care initiative. Specifically, it aims to identify the factors—both enablers and barriers—that impact the integration among organizations within this service.

## Methods

### Design

This study is a qualitative process evaluation using semi-structured interviews. Process evaluation looks at all of the components of an intervention - the processes, the people involved, the environment, etc. - to determine what worked and what did not [63]. In the context of healthcare interventions, qualitative process evaluations can provide deeper insights into the processes involved, offering explanations for the success or failure of initiatives. They are crucial for understanding and developing complex interventions, assessing fidelity, reach, implementation barriers, and participant experiences, thereby optimizing these initiatives [64–66]. Additionally, qualitative evaluations offer insights into intervention processes, feasibility, acceptability, and opportunities for improvement [67]. They are instrumental in exploring healthcare professionals’ and patients’ perspectives on interventions, understanding intervention components, and assessing variations in impact among different subgroups [68]. By conducting semi-structured interviews, this study aimed to gather in-depth information from individuals involved in the service delivery, allowing the identification of areas that work well or require improvement in the service.

Through semi-structured interviews, this study aimed to collect detailed information from individuals involved in service delivery, identifying effective areas and those needing improvement. We explored the experiences and perceptions of healthcare professionals on the integration of healthy lifestyles and mental health services of TWL. The consolidated criteria for reporting qualitative research (COREQ) checklist [69] were completed to ensure all relevant items for reporting qualitative research were included (Supplementary file 1).

### Study setting

Luton is a Borough situated in Bedfordshire, South East England. It has a population of 213,500 [70]. Luton is ranked 70th most deprived area in England out of 317 local authorities [71]. In Luton in 2020/21, 67.5% of adults were overweight or obese, as opposed to 63.5% for England [72]. The premature mortality in adults with severe mental illness in Luton has higher rates than the England and regional average values and the life expectancy in Luton is lower than the England average [73].

The service under evaluation is the ‘Total Wellbeing Luton’ (TWL), an integrated and proactive care pathway for physical and emotional health available to people who live or are registered with a primary care provider in Luton, UK. Turning Point and Active Luton are the health and care organisations, that partnered to provide Improving Access to Psychological Therapies (IAPT) and healthy lifestyle services, including weight management programmes, exercise on referral, social prescribing, and smoking cessation, respectively. The purpose of the service is to build an integrated and proactive continuum of care for people who live in Luton, UK.

### Participants and sampling

This study aims to explore the perceptions and experiences of healthcare professionals concerning the integration of healthy lifestyle and mental health services of TWL. The potential participants needed to be service providers of TWL, who had experience in implementing and delivering interventions. Therefore, a purposive sampling technique was used to identify and select participants based on their ability to contribute narratives that enable an in-depth understanding of the research problem [74, 75]. Based on the purposive sampling strategy, the following key service providers groups were identified as appropriate for semi-structured interviews in the study: Senior leadership [3], Service managers [4], Healthy lifestyle specialists [7], Psychological Wellbeing Practitioners [5] and Wellbeing Coordinators [1]. By interviewing individuals from these specific groups, a comprehensive range of perspectives would be gathered about the factors that influence the integration among the participating organisations. A suggested sample size between 12 and 20 individuals overall is considered appropriate for research that aims to investigate a research question from a stakeholder perspective [76]. Therefore, a sample of 20 participants can be considered a sufficient sample size to explore service providers’ experiences and perceptions.

Thematic saturation was reached by the 9th interview. Saturation means that no additional data are being found and is commonly used as a criterion for judging whether data collection should be continued or not in qualitative research [77]. As the researcher encounters similar patterns repeatedly, the researcher becomes empirically confident that a category is saturated [78, 79]. Thematic saturation was achieved through the continuous integration and comparison of new data with the existing thematic framework. This process involved actively seeking out new themes and variations while critically assessing the comprehensiveness of current themes. The iterative nature of the analysis ensured that data collection continued until no new themes or significant insights emerged from subsequent interviews, indicating that thematic saturation had been reached. Consequently, the recruitment of the participants stopped when saturation of the data was reached.

### Data collection

The principal researcher (FL), a PhD candidate, with experience in qualitative research, conducted all the interviews face-to-face between November 2019 and March 2020. The interviews with the service providers were conducted face-to-face in TWL premises. Before the interviews, the participants were fully informed about the process of the interviews. They were informed that the interview would be audio-recorded using the researcher’s personal audio recorder. The length of the recordings was between 20 and 45 min. The interview schedule was developed after reviewing the existing literature to gain an understanding of the experiences and perceptions of service providers on the integration of the services provided by TWL (Supplementary file 2). The drafted interview schedule was discussed and agreed upon with co-authors and funding partners, who have research and administrative experience in healthcare.

### Data analysis and interpretation

The interviews were audio recorded and transcribed verbatim. The transcripts were imported into the computer-assisted qualitative data analysis programme NVivo 11 to facilitate data analysis. To ensure the principles of anonymity, any information, which may reflect the identity of the participants, is not included. All transcripts were anonymised, and each participant was ascribed as ‘participant’ and a number, e.g., participant 1.

Generic qualitative research approach and thematic analysis (TA) were applied to all interviews through a structured six-phase process, proposed by Braun and Clarke [80] ensuring methodological consistency and analytical depth. The phases of the TA applied in this study are the following: familiarisation with the data, generating initial codes, generation of themes and categories, reviewing codes, sub-themes, and themes, data interpretation and presentation, and write-up. The familiarization phase entailed repeated readings of the transcripts, allowing researchers to immerse themselves thoroughly in the data. The principal researcher (FL) independently conducted coding to identify meaningful data segments. A member of the team (YP) coded 20% of the transcripts. Regular team discussions facilitated the iterative refinement of these codes, leading to the development of preliminary themes. These themes were systematically reviewed against the entire data set to ensure they accurately represented participants’ experiences and were coherent and distinct. Detailed notes and reflexive memos were maintained throughout the process to document analytical decisions and reflexive insights, thereby enhancing transparency and rigour.

Generic qualitative research is an approach that “seeks to discover and understand a phenomenon, a process, or the perspectives and worldviews of the people involved” [81]. It is a flexible approach designed to explore experiences and perspectives within a specific context. This flexibility allows researchers to focus on producing descriptive, context-rich insights tailored to the specific phenomena under investigation. Similarly, TA is a flexible, interpretative approach to qualitative data analysis that assists in the identification of themes in a given data set [82, 83]. The process of coding and developing themes aimed to incorporate both descriptive and interpretive elements [83]. The descriptive aspect sought to capture participants’ statements, while the interpretive component utilized researcher’s subjectivity to identify less obvious patterns, such as those influenced by social context. Employing an interpretive approach, the lead investigator delved into the participants’ descriptions, uncovering themes and connections within their experiences. This analysis subsequently facilitated the alignment of these findings with the established body of relevant literature.

Reflexivity is an important part of ensuring the trustworthiness of qualitative research [84]. The credibility of the study was ensured using reflexive notes. Reflexivity has been applied to acknowledge incompleteness and take responsibility for the researcher’s perspectives [85]. In conducting this study, the research team maintained reflexivity to enhance the trustworthiness and credibility of the findings. Reflexive notes were taken throughout the research process, allowing the researchers to document and examine their own perspectives, assumptions, and experiences as they engaged with the data. This reflexive practice served as a tool to critically assess the influence of personal backgrounds and potential biases on data interpretation. The research team acknowledges that their individual backgrounds - comprising diverse professional training, cultural perspectives, and previous research experiences - may have shaped their initial assumptions and focus. By openly reflecting on these aspects, the researchers were able to recognize and address any biases that might impact the study’s design, data collection, and analysis.

### Ethics approval and consent to participate

A favourable ethics opinion was obtained from the Institute for Health Research Ethics Committee (IHREC) of the University of Bedfordshire, UK (IHREC925). All participants signed a written form of consent after having received oral and written information about the study.

## Results

The thematic analysis identified five themes which provided an understanding of the experiences and perceptions of the service providers on the integration of healthy lifestyle and mental health services of TWL. Each theme was divided into sub-themes. The following themes were identified:* 1) Culture, 2) Communication structures, 3) Resources and 4) Expectation and reality of the integration. *To assist the visualisation of the themes/sub-themes, a coding tree is provided as Supplementary file 3. Additionally, a theme standardization table was developed (Supplementary File 4), categorizing the sub-themes as enablers and barriers that affect the integration among organizations within the service. Each of these themes are discussed in more details below.

### Theme 1: Culture

#### Relationships between professionals

Collaborative working was perceived as a primary strength of the service. Participants highlighted that they do not work in silos, within organisations. All participants commented on the excellent working relationships, which in line create a good working environment where staff are happy to work.

*“I would say the teamwork and the team within all services, we work so close together, and we get on with each other so well, that it makes the service, so much better. We're always really happy to come into work.” (Participant 7)*

*“We don't kind of work in silos. Everyone helps each other out, which is great.” (Participant 16)*

#### Personal interest and motivation

Staff motivation and engagement appeared as an enabler to integration. It was reflected that members of staff are passionate about their job, they truly believe in the service’s mission and they try to pass “their passion” to the service users to achieve the best possible change.

“I know everyone is so passionate about what they do, as well, which makes a big difference, especially when you're like promoting it to people who are a little bit reluctant to change. So, I'd say that's our biggest strength.” (Participant 16)

In addition, participants expressed that they are proactive, and they want to collaborate with other teams in order to do their job, as the best they could.

And also, I've spoken to the IAPT team, because I wanted to go and sit with them to see how their service work. …. I am quite proactive. So, I've asked to go and work with them. I am quite proactive I like to indulge in different areas.” (Participant 4)

While high levels of personal interest and motivation were reflected from healthy lifestyle staff, IAPT staff reflected that they are focused on their job, as the workload does not allow them to engage with other teams.

*“I guess when we're just with patients all day or in an office you don't see it as much because you're just focused on doing your job. Just getting as many clients seen as possible.” (Participant 12)*

#### Lack of inter - organisational collaboration

Participants expressed the view that the healthy lifestyle team and the mental health teamwork in a non-integrated way. There have been organised integrated days to encourage a collaboratively way of working but it is not such successful, as it should be.

“I feel is healthy lifestyle and the emotional health are totally separate. And we try…. It's difficult. We've done a number of integrated days and things like that. And but I wouldn't say that they've been very successful.” (Participant 16)

Participants also stressed that the workload does not allow them to book a whole day in their diary for joined events and integrated training.

*“My diary is so full, is very hard to take a whole day out of my diary. […] It is not a meeting; it is like to get to know the other. It is a good thing but a whole day is difficult.” (Participant 3)*

### Theme 2: Communication Structures

#### Lack of shared IT system

Service providers repeatedly expressed their concerns about the poor functionality of their information systems to transfer service users’ records across settings. Each organisation uses a different information system. The lack of a joined-up network across organisations was acknowledged as a barrier to service users’ data sharing and cross-referral processes between the services.. Participants revealed that the lack of an electronic system accessible across settings impedes the integration between the organisations, as it does not facilitate the communication and information flow.

“One of the challenges is to make sure that the information is passed. One of the things that has made that difficult is the databases that sit behind. So, in an ideal world, to have one database, one system that sat behind everything we do, because at the moment, the talking therapy staff are Turning Point employed and use IAPTus and then the healthy lifestyle service staff, who are employed by Active Luton, use DCRS. So, there's some structural things in there that make it more difficult, that all that go against the integration.” (Participant 16)

Participants, also, presented their views on why there is a lack of one IT system for the collaborating organisations. The organisations are commissioned independently to provide emotional and physical health. Therefore, the lack of pool funding cannot support the use of one IT system, which is tailored to the needs of the service, as a whole. Participants stressed that the different IT systems, “do not talk to each other”, which is an important barrier to the integration.

*“We have two different systems, and they do not talk to each other. So that's the barrier. For the integration and it will always be a barrier, because we can't physically change the systems, because IAPTus has been commissioned for the emotional health and DCRS has been commissioned for the physical health, for the duration of our contract.” (Participant 18)*

#### Absence of physical Co-location

The lack of a shared setting is an important barrier of the integration of the services. Teams are located in different settings, which impedes the communication and the collaboration between staff members.

*“It is a massive barrier to people. […] I've heard team members say we're not all in one building so how can we work together.” (Participant 18)*

According to participants’ accounts, the initial plan was for the teams to be located in the same setting. However, it was a priority to offer a location-flexible service in order to be easily accessible from service users across Luton. This is contradictory to the initial plan; to host all the services in one setting.

*“From the location perspective, I always thought that the staff will be based in the same place. [….] It's not really coordinated in that way in the moment and so I'm thinking from an admin and a base perspective you would have staff this in the same place.” (Participant 16)*

### Theme 3: Strategic alignment

#### Lack of integrated commissioning

Participants expressed that there is lack of an integrated commissioning framework. It was mentioned that the funding bodies fund either the IAPT service, either the healthy lifestyle service, but they do not meet with each other, except during the commissioners meeting. Participants raised the issue that the service appeared as two different services to all stakeholders, including service users. Interviewees clearly stated that the funding and the interest of the funding bodies are dichotomized. Participants expressed that there is a “*natural split in the service from the very top”, and *believed that commissioners do not perceive the service as a whole, but as two separate parts. It was stated that an integrated commissioning would facilitate the integration of the service.

*“I think the best-case scenario is that people is just seen us as one service. […] And I think comes from all the way at the top, because it’s commissioned by Luton CCG and public health. And then Luton CCG have their interest in IAPT and Public Health have their interest in healthy lifestyles. So even from the very top, there’s a natural separation. It is happened from the very top, so CCG and public health needs to be more integrated when it comes to TW” (Participant 14)*

#### Branding for cohesive identity

Branding in healthcare is the process of shaping how a healthcare organisation is perceived. The following quote shows that staff perceived the service as two separate services and not as an integrated whole.

“What's it supposed to be, I understand it is one service, but it's effectively two services.” (Participant 1)

Participants reported that the logo or the name of the organisation on the paperwork reflect that there two different organisations, who run the services for TWL. Participants also reflected on how this perception affects the integration between the two organisations and how the service is perceived by the community. Service providers expressed the concern that “small” branding tags on paperwork, uniform or badges create confusion to stakeholders and to the public.

*“I'm still going along to meetings, GPS are saying… you’re Active Luton and I'm TW, they're like two separates… it's still very confusing for people. Because even, name tags, I've got TW here… even that is confusing. Our uniform says Active Luton and TW. It sounds silly but those small things do make a big difference. And from what people can see the service. So, I think when you talk about integration Active Luton and Turning Point needs to set aside and its TWL that is the integrated service.” (Participant 19)*

#### Capacity building for sustainable collaboration

Lack of integrated training appeared as a barrier to integration. Participants expressed the view that integrated training may be beneficial in understanding everyone’s role in the service and improve the integration between the two organisations.

*“There was like I think a week of training where we all kind of got together and that was really nice because you got to meet the other, the other team members and kind of maybe understand their role a little bit more.” (Participant 11)*

Some comments reflect that there is a lack of induction training which the participants expected to have existed.

*“I think, a proper induction program would really help. An induction that involves both sides of the service. It includes healthy lifestyles, includes IAPT, it includes what TW is,” (Participant 14)*

### Theme 4: Workforce dynamics

#### Recruitment and retention

Participants also expressed their thoughts on the impact of temporary agency staff on the integration between the teams. It was highlighted that the agency staff are not committed to service. From the quote it appears that the nature of the short employment contracts is linked with the lack of investment and commitment to the service.

“There is a high percentage of agency staff. And agency staff is just whether are they fully invested and committed to TWL or are they just because it’s the latest contract they’ve got and for the pay check rather than actually invested in and feeling part of the service.” (Participant 14)

Managers and staff expressed the view that a high staff turnover had effect on the collaborative working between the different teams. When they asked on what they think does not work well in the service, they highlighted the high staff turnover issue.

*“Because people move around so much, I lose track. It is mainly me, my fault not keeping up with who is moving where.” (Participant 3)*

*“But there has been quite a lot of staff turnover, which doesn’t help.” (Participant 14)*

#### Shared leadership

Participants expressed their optimistic view about the new role of integration manager. They reported that the new role is critical for the improvement of the integration between the two participating organisations. Staff recognised that they tend to split the services and stress the importance of a central integration manager, who will overlook the service as a whole.

*“She's just started in it he's going to be looking at how, how we achieve that integrated approach and see the service as a whole, rather than as a particular program, because we tend to split healthy lifestyles and IAPT for many different reasons. Her job is to look at how we keep things connected as much as we can.” (Participant 13)*

#### Navigating workload challenges

Participants stated that understaffed areas in the service may increase the workload. Interviewees also reflected that staff feel overwhelmed by their workload and this in line lead to increased staff turnover.

*“But I think sometimes people can get overwhelmed by how much work they've got. And because there isn't enough staff always, and then you've seen several people leave. And then you've got to retrain people and stuff.” (Participant 17)*

### Theme 5: Expectation and reality of the integration

#### Under one roof

In the initial implementation of the TWL it was envisioned to create an integrated service, where people who live in Luton can access and receive support to improve their physical and mental health. When interviewed, most of the participants understood that TWL is an integrated service, as it offers physical and emotional support, “under one roof”. There was an expectation that the service would provide physical and mental health services under one “umbrella”. Participants confirmed that this expectation meets the reality of the implementation of the service.

*“Ensuring that the clients get the best possible care for both their physical and emotional health at the time that's right for them. So, I see it that a client should come into TWL for any health behaviour change of their choice, and then be able to move with it across the services seamlessly and safely to make sure that they get the best service to meet different health needs.” (Participant 15)*

#### Single point of access

Wellbeing coordinators’ role is to offer a single point of access to the service. Wellbeing coordinators receive referrals, complete holistic assessments, and book potential service users in sessions. Participants acknowledged the difficulties of meeting the expectation of a single point of access. They referred to wellbeing coordinators’ capability and the fact that health care professionals are referring people directly to health specialists, without accessing the wellbeing coordinators. From the following quote, it seems that the expectation of a single point of access in the service does not meet the reality.

*“The theory behind it that it was a single point of access. That is probably a bit blurred. Some health professionals, specifically around the hospital secondary care refer directly to members of staff. So instead of going through a single point of access. So, some professionals have kind of lost their trust in that single point of access, where people were getting referred into it and then their patients weren’t hearing anything, or the response wasn’t timely. So, I think that element hasn’t worked as well as it could do.” (Participant 13)*

#### Service users “tell their story once”

The expectation of TWL was for the service users to “tell their story once”; and provide their health-related information once. Although, in reality, this is appeared to not be the case. Participants stated that service users need to repeat information that should not be repeated, such as sociodemographic information. When interviewed and asked if the service users need to tell their story more than once, participants clearly stated:

*“I think people should only give their information once, particularly things like their age, date of birth, ethnicity, and they will need to repeat certain things that we are monitoring in terms of changes, but I think for as much as can be just asked once should be… this certain information that shouldn't be repeated.” (Participant 13)*

## Discussion

This study aimed to understand the experiences and perceptions of healthcare professionals and stakeholders on the integration of the healthy lifestyle and mental health services of TWL. Using thematic analysis, we explored the experiences and perceptions of service providers. The analysis of the interview transcripts revealed five main themes: 1) Culture, 2) Communication structures, 3) Resources, 4) Workforce dynamics, and 5) Expectation and reality of integration. The identified themes were grouped as enablers and barriers to the integration, and their interrelationships were discussed.

Interdisciplinary teamwork and staff engagement were perceived as important factors that facilitate the integration between the organisations. This aligns with previous studies, where the emphasis was on collaborative working, professional engagement, and shared values and understanding, as enablers of the implementation of integrated care initiatives [38, 86–88]. It was also found that the employment of bank staff, which is responsible for high rates of staff turnover, affects team dynamics. Consequently, the post of an integrated manager is crucial in inter-organisational integration. The new role of an integrated manager in TWL creates an optimistic view of better integration between the teams. This is in good agreement with previous studies, which have shown that committed leadership may work in favour of inter-organisational integration through sharing a vision of integration with their employees [38, 89].

In TWL, the lack of a shared Information Technology (IT) system was identified as the key barrier to integration. Different IT systems impede information transfer, communication, and efficient cross-referral processes between the teams. These findings are in line with previous research on key stakeholder experiences of integrated healthcare pilots [87, 90]. The findings of the current study are also consistent with other research which found that incompatible IT systems affect the level of inter-organisational integration in integrated healthcare initiatives [89, 91]. The lack of a shared IT system not only hinders collaboration between different teams within TWL, but it also affects the overall effectiveness and success of integrated healthcare initiatives. Without a seamless way to transfer information and communicate between organizations, the potential benefits of integrated care may not be fully realized. Addressing the issue of incompatible IT systems is crucial in promoting successful integration and improving patient outcomes in healthcare settings. Consequently, improved communication and exchange of health records among various organisations may result from an IT system that is accessible to all of them. This could significantly enhance the overall coordination of care and improve patient outcomes. By having a shared IT system, healthcare providers can easily access and update patient information, leading to better-informed decisions and more seamless transitions of care. Ultimately, investing in compatible IT systems can help break down silos between healthcare organizations and promote a more integrated approach to patient care.

This study highlights how the lack of shared IT systems and co-location, affects the communication between staff across organisations. An integrated IT system will achieve a direct and effective information transfer across organisations and therefore enhance communication. This is also supported by the available literature [86, 92, 93]. Interestingly, an extensive part of the literature supports that one of the main facilitators to improve communication is co-location [86, 87, 93–97]. Studies have specifically shown that teams who are based in the same setting are more likely to collaborate, even if it is an informal conversation [86, 94, 96]. As a result, the study’s findings emphasise how crucial co-location and shared IT infrastructure are to employee collaboration across organisations. They suggest that physical proximity plays a significant role in fostering effective communication and collaboration among employees from different organisations. Therefore, organisations should consider implementing strategies to promote co-location and provide a shared IT system to enhance cross-organisational collaboration and communication.

An interesting theme that has been revealed from the interviews is the “Expectation and reality of the integration”. While the initial implementation plan of the TWL was envisioned to be an integrated healthcare service that offers physical and emotional support “under one roof” to Luton residents, accessing through a “single point of access” and “tell their story once”, the reality was revealed to be different. For example, a “single point of access” was envisioned, as the central hub, which receives all the referrals from the community or self-referrals, and as a way of conducting an initial assessment of potential service users; and assess their eligibility for an intervention. The “single point of access” was planned to be a facilitator of the integration between the teams. This is in good agreement with Brown et al.’s (2003) study, which states that a “single point of access” improves communication between teams and understanding of team member’s roles, reduces the assessment time, and increases the number of self-referrals [98]. To some degree, the “single point of access” has been achieved in TWL. Therefore, the “single point of access” has been implemented, but still, there are referrals, which come directly from the third party to TWL teams.

Furthermore, the service was envisioned to offer physical and emotional support “under one roof”. This study found that the “umbrella” brand does not necessarily ensure coordinated care, as the participating organisations continue to work in a siloed manner. This finding highlights the importance of branding in integrated health services. Participants expressed how branding defines the service. For service users, the brand TWL reflects what the service is about; a service that supports both emotional and physical health. However, service providers perceive the service as two different organisations, because of how they refer to “themselves”: as Turning Point or as Active Luton. In addition, details such as a brand logo on the paper, professional badges with logo, and different email domains have been identified as barriers to perceiving the service as a whole. In the literature, there seems to be an agreement with the present findings, as Keeling et al. (2018) argue that much attention is focused on the implementation and design of processes, rather than focussing on how services are presented to service users and service providers [99]. Also, current literature has been published on the role of marketing as an integrator in the context of integrated care [99, 100]. It is suggested that marketing can act as a lynchpin between interdisciplinary teams, providing clarity to service users and providers. This is based on evidence that marketing has a notable track record of effectively organising multidisciplinary branches for a common goal [100–102]. These findings indicate that a clear, widely agreed branding for service providers may influence positively the organisational integration of TWL.

The study sheds light on the dynamics influencing the integration of healthy lifestyle and mental health services within the TWL initiative. The identified themes unveil the complexities faced by healthcare professionals and stakeholders and reflect factors that facilitate or impede the integration between the participating organisations. This study found interrelationships between the identified factors that facilitate or impede the integration in TWL. For example, a common view amongst service providers was that bank staff are not committed to the service, potentially due to a lack of career prospects, which then leads to high staff turnover rates. Increased staff turnover creates the need for new staff which then necessitates further training to develop staff readiness and time to rebuild staff relationships and shared understanding. Therefore, one factor which works as a barrier to integration triggers other factors. This interconnected web of factors highlights the complexity of integrating TWL services. It becomes evident that addressing one issue, such as staff turnover, requires a comprehensive approach that considers the broader context in which this challenge arise. Ultimately, fostering a culture of commitment and career advancement opportunities for bank staff may help mitigate the negative effects of high turnover rates and pave the way for successful integration of TWL services.

Throughout the interviews, facilitators of the integration have been identified, such as sharing leadership and shared IT systems across the organisations. These facilitators have also been identified in the literature, as mechanisms for achieving types or dimensions of integration [27, 103]. For example, a higher level of integration may be achieved through shared IT systems and recruitment of an integrated manager (organisational integration), better communication/information transfer (functional integration), and staff readiness (professional integration). The importance of staff engagement and personal motivation has also been reported as a requirement for integration (normative integration). In the literature, it has appeared that these types of integration are needed to achieve an overall effective integration [17, 20, 27].

In the literature, it appears that the collaboration between health and social care is ineffective. The current models are unable to tackle the challenges they currently confront because they are inflexible, linear, and restrictive to innovation [104]. With the passing of the Health and Care Act 2022, the NHS is seeing the biggest reforms in nearly a decade. This is because it unifies NHS, social care, and public health services at a local level and aims to tackle a growing health inequality problem. At the heart of the changes brought about by the Act is the formalisation of integrated care systems (ICSs). ICSs are designed to break down the barriers between health and social care by promoting collaboration and coordination among different service providers. By bringing together healthcare professionals, social workers, and other stakeholders, ICSs aim to improve the overall quality of care and address the complex needs of individuals more holistically. Additionally, the Act emphasizes the importance of patient involvement and empowerment, recognizing that their active participation is crucial for the success of integrated care [105]. The findings of this study could contribute to the current policy discourse on workforce development, organizational commitment strategies, and the importance of addressing systemic issues affecting the integration of health services within broader initiatives like TWL. Service providers may find value in considering these interrelated factors when designing and implementing initiatives aimed at enhancing the integration of health services within the workplace.

This research has significant implications for policy and service practices. The present study is one of the first studies evaluating the integration between the participating organisations of an integrated service for physical and mental health in the UK. Specifically, it identifies factors that influence the integration between the organisations enabling decision-makers, contract holders, and managers to promote positive influential factors and overcome barriers to improve the implementation of integrated healthcare services. In addition, this research offers an overview of the practicalities of implementing an integrated care initiative. Structures, people, values, and processes need to be considered as elements of a whole system and not in silo. This study examines how organizations can effectively integrate physical and mental health services, emphasizing key factors essential for successful collaboration. It underscores the importance of cohesive systems, shared values, and clearly defined processes to enhance service delivery. Key findings underscore the benefits of a unified IT system to facilitate communication, a consolidated health budget for efficient resource allocation, and a dedicated professional body to support staff development and retention in integrated care. Co-location of services and active stakeholder engagement are also identified as critical strategies for fostering stronger collaboration.

The study provides actionable recommendations for healthcare administrators and policymakers.

Administrators should establish structured multi-agency meetings, develop shared performance metrics, implement staff training programs, and facilitate data-sharing agreements to enhance interoperability. They should promote cross-sector collaboration through regular multi-agency meetings and shared performance metrics. Policymakers are advised to mandate integrated commissioning for sustainable funding, enforce interoperability standards for health IT systems, and formulate national workforce policies that facilitate career advancement in integrated care. Additionally, policymakers should incentivize service co-location through targeted funding and establish engagement frameworks that integrate service user feedback into the design of integrated care services.

Based on the findings of the study, future research should explore several key areas to further enhance inter-organizational integration in integrated care initiatives. Longitudinal studies are needed to track the evolution of integration over time, examining how relationships, structures and strategies develop and adapt. Comparative analysis of different integrated care initiatives across regions or countries can help identify best practices and common challenges. Additionally, studies should focus on the involvement of a broader range of stakeholders, including commissioners, and service users, to understand their perspectives on integration. Addressing these areas will provide a comprehensive understanding of the complexities involved in inter-organizational integration within healthcare and inform the development of more effective integrated care models.

### Strengths and limitations

This study was conducted among managers, health care professionals, senior leadership team, and coordinators working in TWL. One of the strengths of this research is the representative sample of interviews (*n*=20), considering the size of the service, with a wide range of healthcare professionals involved in the daily delivery of the interventions and managers. In addition, the participants were interviewed during the second year of the service’s operation, which offers insights on the areas where changes need to be implemented to improve the level of integration between the participating organisations in TWL.

Finally, a number of important limitations need to be considered. We acknowledge that the conclusions and insights derived from your study, which are primarily centered on the perceptions and experiences of staff members, may not be broadly applicable to all stakeholders involved in the implementation of this initiative (e.g. commissioners). This limitation means that the findings are not representative of the perspectives and experiences of commissioners or other groups of stakeholders beyond the staff.

Another limitation involves the generalizability of the findings. Qualitative research often focuses on context-specific insights that may not be directly applicable to other settings. The unique characteristics of this study’s setting may limit the applicability of the findings to different environments or organizations with varying structures, resources, or stakeholder dynamics. Thus, caution should be exercised when applying these insights to other contexts.

Lastly, there are methodological constraints to consider. The reliance on self-reported data introduces potential biases, as participants’ responses may be influenced by factors such as social desirability or recall bias. Additionally, the use of reflexive notes, while enhancing credibility, also introduces the potential for subjective interpretations by the researchers. Although reflexive practices were employed to mitigate these effects, the influence of researchers’ perspectives cannot be entirely eliminated.

## Conclusion

This qualitative study aimed to understand the experiences and perceptions of service providers to inter-organisational integration in the TWL context. The findings illustrate different factors (e.g., relationships between healthcare professionals, communication structures, financial resources, human resources) that influence the integration between the participating organisations of TWL. These factors are independent but interconnected with each other. For example, the lack of functional integration (e.g., communication structures) affects organisational integration (e.g., inter-organisational collaboration). These findings highlight the critical role of shared IT system and leadership across the organisations, organisations’ culture, and resources in enhancing inter-organisational collaboration within integrated care initiatives. In addition, the study provides an overview of how the service was envisioned and what the reality is. This in line suggests that the need for collaboration between stakeholders involved in decision-making, and allocating funding, is more pronounced now than ever.

As the UK seeks to embed 42 Integrated Care Systems (ICSs) since their establishment in July 2022, this study provides a timely insight into the challenges and opportunities provided by the ambition of inter-organisational integration. Other countries are embarking on the journey of integration too, so the findings have relevance to other contexts too. Our study highlights that there is no ‘one-size fits all’ approach to inter-organisational integration. Indeed, there are many factors that impact the success or failure of integration approaches, and it is important that Governments and policymakers are cognisant in creating an organisational development culture that seeks to support the successful journey to inter-organisational integration.

## Supplementary Information

Supplementary Material 1.

Supplementary Material 2.

Supplementary Material 3.

Supplementary Material 4.

## Data Availability

The datasets generated in this study are not publicly available due to sensitive and personal nature of the information. Data may be available upon request to the corresponding author (FL), with restrictions following ethical approval.

## Abbreviations

CAS: Complex Adaptive System
IAPT: Improving Access to Psychological Therapies
ICS: Integrated Care System
IT: Information Technology
NHS: National Health Service
TA: Thematic analysis
TWL: Total Wellbeing Luton
WHO: World Health Organisation
