# Supplementary material for: Factors that influence inter-organisational integration: a qualitative exploration of service providers’ perspectives from an integrated care initiative
Source: BMC Health Serv Res. 2025 Jul 10;25:947. doi: 10.1186/s12913-025-13051-7 (PMC12247228; doi:10.1186/s12913-025-13051-7)
Supplement: Supplementary file 2 — Supplementary Material 2. [file 12913_2025_13051_MOESM2_ESM.docx]

**Supplementary file 2**

**Interview schedule for service providers**

**Introduction**

- Thank participants for agreeing to take part in this interview.
- Provide background to the project: I am interested in your perceptions and experiences.
- Brief outline of the interview: We will discuss about your perceptions of the service and its impact. There are no right or wrong answers.
- Explain about the consent procedure, highlight that the interview will be recorded, they do not have to answer any question they do not want to, stress that the participation in voluntary and they can stop the interview any time without giving any reason.
- The findings from the research will be written up for my Ph.D. thesis and may be published in academic / professional journals.
- Confidentiality. Any quotes will be anonymised and I will ensure that there are no issues than can trace the interview with them.
- Before we start, participants will be asked to sign two copies of the consent form. One of them and one for the project’s file.
- Any questions or concerns before we start the interview?

**Understanding service provider’s role in the service**

- Can you tell me about your role in the Total Wellbeing Luton?

**Knowledge – Total Wellbeing**

- How would you describe the Total Wellbeing Luton?
- Where do you think patients that use Total Wellbeing hear about the service?
- How does the Total Wellbeing Luton service works in practice?

**Understanding provider’s perception of integrated services**

- Total Wellbeing Luton has been described as an integrated healthcare service. How do you understand the term “integration”?
- How can you describe an integrated healthcare service?
- From your experience, how do you understand the integration in the Total Wellbeing service?
- *If not covered*: How do you understand the integration between health lifestyle services and IAPT service?

**Understanding providers’ experiences of integrating the services**

- What criteria do you use to identify patients who need to be referred to another service?
- Do you refer patients to other services on a regular basis?
- How do you refer the patients to another service?
- Can you describe the communication between the services during the cross-referral process?

**Understanding the data collection process**

- How do you measure the outcomes of the service? What tools / measurements do you use?

**Understand service providers’ perceptions of patients’ outcomes evaluation**

- How often do you follow up the patients?
- From your experience, what are the outcomes of patients after the completion of the intervention received by Total Wellbeing Luton?
- From your experience, what are the outcomes of patients that you have referred to another service?
