# Supplementary material for: Factors that influence inter-organisational integration: a qualitative exploration of service providers’ perspectives from an integrated care initiative
Source: BMC Health Serv Res. 2025 Jul 10;25:947. doi: 10.1186/s12913-025-13051-7 (PMC12247228; doi:10.1186/s12913-025-13051-7)
Supplement: Supplementary file 3 — Supplementary Material 3. [file 12913_2025_13051_MOESM3_ESM.docx]

# Theme standardization table

| **Theme** | **Subtheme** | **Enablers** | **Barriers** |
| --- | --- | --- | --- |
| **Culture** | **Relationships between professionals** | Strong relationships trust, open communication |  |
|  | **Personal interest and motivation** | Shared passion for IC, proactive approach to collaboration | IAPT staff workload limits collaboration |
|  | **Lack of inter - organisational collaboration** |  | High workloads, unsuccessful integrated days |
| **Communication Structures** | **Lack of common platform** |  | Separate IT systems, lack of unified data sharing, lack of funding for a common platform |
|  | **Absence of physical Co-location** |  | Teams located in separate locations, hindering collaboration |
| **Strategic alignment** | **Lack of integrated commissioning** |  | Separate commissioning frameworks, fragmented service structure |
|  | **Branding for cohesive identity** |  | Disjointed branding, confusion among stakeholders and service users |
|  | **Capacity Building for Sustainable Collaboration** |  | Lack of comprehensive induction and integrated training programs |
| **Workforce dynamics** | **Recruitment and Retention** |  | High turnover, reliance on temporary staff |
|  | **Shared leadership** | Introduction of an integration manager |  |
|  | **Navigating workload challenges** |  | Understaffing, high workload, staff burnout |
| **Expectation and reality of the integration** | **Under one roof** | Centralized location offering both physical and mental health services |  |
|  | **Single point of access** |  | Confusion about access points, healthcare professionals bypassing the single point of access |
|  | **Service users “tell their story once”.** |  | Repetition of information due to incompatible systems |
