# Supplementary material for: Factors that influence inter-organisational integration: a qualitative exploration of service providers’ perspectives from an integrated care initiative
Source: BMC Health Serv Res. 2025 Jul 10;25:947. doi: 10.1186/s12913-025-13051-7 (PMC12247228; doi:10.1186/s12913-025-13051-7)
Supplement: Supplementary file 4 — Supplementary Material 4. [file 12913_2025_13051_MOESM4_ESM.docx]

**Supplementary file 3**

**Coding tree - Factors that influence inter-organisational integration: a qualitative exploration of service providers’ perspectives from an integrated care initiative**
